# Risk factors of grade ≥ 2 radiation pneumonitis after gemcitabine induction chemotherapy for patients with non-small cell lung cancer

**DOI:** 10.1186/s13014-019-1440-8

**Published:** 2019-12-16

**Authors:** Liming Sheng, Xiaoying Cui, Lei Cheng, Ying Chen, Xianghui Du

**Affiliations:** 1 Department of radiotherapy, Cancer Hospital of University of Chinese Academy of Sciences, Zhejiang Cancer Hospital, Hangzhou, China; 2 Institute of Cancer Research and Basic Medical Science of Chinese Academy of Sciences, 1 Banshandong Road, Hangzhou, 310022 Zhejiang China; 3 The Second Clinical Medical College, Zhejiang Chinese Medical University, Hangzhou, China

**Keywords:** Non-small cell lung cancer, Radiation pneumonitis, Risk factor, Dose-volume variable

## Abstract

**Objectives:**

To observe the risk factors affecting the occurrence of RP after gemcitabine-based induction chemotherapy.

**Methods:**

Between January 2010 and December 2017, patients with NSCLC received gemcitabine or docetaxel chemotherapy, followed by radiotherapy at Zhejiang cancer hospital were enrolled in this study. Patients were treated with gemcitabine or docetaxel induction chemotherapy, followed by radiotherapy or concurrent chemoradiotherapy. Acute radiation pneumonitis was scored post chemoradiotherapy.

**Results:**

One hundred and eighty-four patients with NSCLC were included in the gemcitabine group and 144 in the docetaxel group. The gemcitabine group experienced a higher incidence of grade ≥ 2 RP, compared with docetaxel group (25.5% Vs. 13.2%, *P* = 0.005). The optimal cutoff values of lung V_5_, V_20_, V_30_ and MLD were set at 44% (AUC [area under the curve] = 0.593), 24% (AUC = 0.607), 14.2% (AUC = 0.622) and 1226 cGy (AUC = 0.626). On multivariate analysis, only lung V_30_ was identified as a predictor for grade ≥ 2 RP (*P* = 0.03). The grade ≥ 2 RP rate was only 9.4% for the low-risk group (Lung V_5_ ≤ 44%, V_20_ ≤ 24%, V_30_ ≤ 14.2%, and MLD ≤ 1226 cGy) in patients received gemcitabine induction chemotherapy.

**Conclusions:**

Gemcitabine chemotherapy before thoracic radiotherapy in NSCLC patients was related to a higher incidence of grade ≥ 2 RP, compared with docetaxel chemotherapy. The Lung dose-volume variable V_30_ was the best predictor of grade ≥ 2 RP.

## Introduction

Concurrent chemoradiotherapy is the standard treatment for locally advanced non-small cell lung cancer (NSCLC) [1]. Historically, the treatment of NSCLC has evolved from radiotherapy alone to combined chemoradiotherapy. It took 40 years for the standard treatment model of concurrent chemoradiotherapy to be established. RTOG9410 clinical trial showed that compared with sequential chemoradiotherapy, concurrent chemoradiotherapy could significantly prolong the median survival time of NSCLC patients, without the increasing treatment-related toxicities [2]. In 2010, a meta-analysis based on six randomized controlled clinical trials showed that compared with sequential chemoradiotherapy, concurrent chemoradiotherapy reduced the risk of death by 16% and increased the 3-year survival rate by 5.7% in patients with locally advanced NSCLC that could not be resected [3]. Although the toxicities of concurrent chemoradiotherapy may be higher and more severe, it does not increase treatment-related deaths compared with sequential chemoradiotherapy. Based on these findings, concurrent chemoradiotherapy is strongly recommended for locally advanced non-resectable NSCLC patients in the current guidelines.

Current guidelines do not routinely recommend induction chemotherapy for patients with non-small cell lung cancer who can receive concurrent chemoradiotherapy, according to the result of CALGB 39801 [4]. However, in patients with large pulmonary tumors or bulky nodal metastasis, induction chemotherapy could reduce the size of the masses so that the radiotherapy plan can be safely performed [5]. Gemcitabine, a cytosine nucleoside derivative, is one of the commonly used drugs in induction chemotherapy of NSCLC [6, 7]. However, it is not recommended for concurrent use with radiotherapy due to its obvious pulmonary toxicity [8, 9]. Even with sequential gemcitabine and radiotherapy, the incidence of radiation pneumonitis (RP) is still high [10]. The understanding of RP has been developed due to the advances in clinical and molecular biology research, but this complication could not be thoroughly avoided. RP seriously affects the quality of life and long-term survival of lung cancer patients [11].

Numerous clinical and dosimetric parameters are associated with RP [12–14]. Dosimetric parameters, including mean lung dose (MLD), V_5_, V_20_, V_30_, are still the best predictors of RP at present [15]. To reduce the risk of RP, the National Comprehensive Cancer Network (NCCN) guidelines limit the dose-volume parameters of lungs as follows: MLD ≤ 20 Gy, V_20_ ≤ 35% and V_5_ ≤ 65%. However, these constraints are based on clinical observations of concurrent chemoradiotherapy for NSCLC. For patients receiving induction chemotherapy, these parameter constraints may be too strict. However on the other hand, for patients who received gemcitabine-based induction chemotherapy and then received thoracic radiotherapy, the restriction might be too loose.

Therefore we conducted this retrospective study to observe whether different chemotherapeutic regimens in induction chemotherapy before radiotherapy affected the incidence of RP. Furthermore, another aim of this study was to observe the factors affecting the occurrence of RP after gemcitabine-based induction chemotherapy. We further used dosimetric factors to generate a predictive model to identify the risk of RP in patients who received gemcitabine chemotherapy before thoracic radiotherapy.

## Methods

### Patients

Between January 2010 and December 2017, 461 patients with NSCLC received gemcitabine or docetaxel chemotherapy followed by radiotherapy at Zhejiang cancer hospital. The inclusion criteria were as followed: Newly histologically or cytologically confirmed NSCLC; clinical stage IIIA or IIIB; inoperable; four cycles of gemcitabine plus cisplatin or docetaxel plus cisplatin chemotherapy before radiotherapy; completion of chest radiotherapy. The exclusion criteria included receiving surgery before or after chemoradiotherapy (*n* = 85) or a total radiotherapy dose of less than 40 Gy (*n* = 48). Finally, 328 patients were enrolled in this study. This study was approved by the Zhejiang cancer hospital institutional review board. Patients’ age, gender, tumor location, histology, TNM stage, chemotherapy regimens, chemotherapy cycles, the interval between chemotherapy and radiotherapy, total radiotherapy dose, and dose per fraction were collected from the medical record.

### Chemotherapy

Gemcitabine or docetaxel was selected as a chemotherapy regimen according to pathological type, economic situation, and personal willingness. Patients were treated with gemcitabine at a dose of 1000 mg/m^2^ in 100 mL of normal saline solution by 30 min intravenous infusion on days 1 and 8 with cisplatin. Docetaxel was administered at a dose of 75 mg/m^2^ by 1 h with standard premedications. Cisplatin was administered at a dose of 25 mg/m^2^ once a day for 3 days. The treatment was repeated every 3 weeks for a total of four courses. The dose of chemotherapy drugs will be modified by a 20% decrease if grade 3–4 chemotherapy-induced toxicities occur. The interval between induction chemotherapy and radiotherapy was about 4 weeks. Radiotherapy could be postponed in case of severe toxicities or poor PS status.

### Radiotherapy

Patients lied down of a wide aperture CT simulator couch in a supine position. A lightweight thermoplastic body mask was used to cover the lower neck, supraclavicular, chest, and upper abdomen. Each patient underwent CT scans ranging from neck, chest, and upper abdomen with intravenous contrast. After scans, the CT images were imported into the Ray-station data management system. Then the gross tumor volume (GTV), clinical tumor volume (CTV), and organs at risk (OARs) were contoured. The OARs included the left and right lungs, esophagus, heart, and spinal cord. The GTV was delineated according to CT images, PET-CT images, and bronchoscope. The CTV was contoured as GTV plus an area bounded by a margin of 5–10 mm depends on the tumor’s pathology. The PTV (Planning target volume) was defined as the CTV plus 5–10 mm to account for the daily setup variation and respiratory movement. Radiotherapy was administered in 2.0 Gy once daily for 5 days per week up to a total dose of 60 Gy. The dose-volume histogram (DVH) was obtained for PTV, lung, heart, and spinal cord. Weekly concurrent chemotherapy with paclitaxel (60 mg/m^2^) and carboplatin (AUC = 2) was administered in some patients if their PS status permitted.

### Assessment of acute RP

Acute radiation pneumonitis was scored monthly after chemoradiotherapy, using the National Cancer Institute’s Common Terminology Criteria for Adverse Events (CTCAE), version 5.0 as follows: grade 1, asymptomatic, clinical or diagnostic observations only, intervention not indicated; grade 2, symptomatic, medical intervention indicated, limiting instrumental activities of daily living (ADL); grade 3, severe symptoms, limiting self-care ADL, oxygen indicated; grade 4, life-threatening respiratory compromise, urgent intervention indicated (e.g., tracheostomy or intubation); and grade 5, death. RP is a comprehensive diagnosis made by radiotherapy oncologist and imaging specialists in combination with radiotherapy history, symptoms, and CT images. The endpoint in this study was grade ≥ 2 RP.

### Statistical analysis

Clinical variables including gender, age, tumor location, ≥2 RP, and tumor stage were tested for differences between the gemcitabine group and docetaxel group by Pearson’s χ^2^ test. DVH metrics such as patient’s total lung volume, PTV volume, lung V_5_, V_20_, V_30,_ and MLD were also analyzed. The discriminative ability of lung DVH parameters (lung V_5_, V_20_, V_30,_ and MLD) was determined according to the receiver operating characteristic (ROC) curves, and the corresponding AUC were calculated. The optimal cut-off point was calculated with an optimal-corrected classified value to provide the best available sensitivity and specificity, based on the Youden index (sensitivity + specificity – 1). The associations between lung DVH parameters and the risk for ≥2 RP was evaluated using unconditional logistic regression analysis. RP-free survival was defined as the time from the last day of radiotherapy to the earliest onset of radiation pneumonitis or last clinical follow-up, where those alive without pneumonitis were censored at the last clinical follow-up date. Survival curves were estimated using the Kaplan-Meier method and compared using the log-rank test. We used the Cox proportional hazards model with the backward selection method for multivariate analysis. All factors with effects ≥2 RP in univariate analysis (P < 0.05) were included in the multivariate analysis. All statistical calculations were performed with SPSS 13.0 for Windows (Chicago, IL, USA). A *P*-value of less than 0.05 was considered statistically significant.

## Results

### Patients’ characteristics

A total of 328 NSCLC patients were enrolled in this study. Clinical, pathological, treatment, and RP characteristics of the patients in the two groups are shown in Table 1. All patients in this study were treated with four cycles of gemcitabine plus cisplatin or docetaxel plus cisplatin followed by thoracic radiotherapy. The study patients had a median age of 62 years (range: 30–77 years). Three hundred patients were male, and 28 were female. Two hundred and forty-two (73.8%) were lung squamous cell carcinoma, 59 (18.0%) were lung adenocarcinoma, and 27 (8.2%) were other types. According to the new IASLC (International Association for the Study of Lung Cancer) staging system, 40 (12.2%), 147 (44.8%), 68 (20.7%) and 73 (22.3%) had T_1_, T_2_, T_3_ and T_4_ disease, respectively. One hundred and 84 were included in the gemcitabine group and 144 in the docetaxel group. Two hundred and fifty-three patients received concurrent chemoradiotherapy, while 75 patients received radiotherapy. There was no significant difference in clinicopathological variables between the two groups (*P*>0.05). The median PTV volume was 324.8 cc in the gemcitabine group and 343.2 cc in the docetaxel group. No significant difference in PTV volume was found between two groups (*P* = 0.504). A similar result was found when total lung volume was compared (*P* = 0.703).

**Table 1:** The relationship between patients’ characteristics and chemotherapy regimens

Variables	N	Gemcitabine	Docetaxel	*P*
Gender				
Female	28	15(53.6)	13(46.4)	0.843
Male	300	169(56.3)	131(43.7)	
Age				
<65	210	112(53.3)	98(46.7)	0.203
≥ 65	118	72(61.0)	46(39.0)	
Tumor location				
Left	145	85(58.6)	60(41.4)	0.434
Right	183	99(54.1)	84(45.9)	
Histology				
SCC	242	140(57.9)	102(42.1)	0.482
Ade	59	29(49.2)	30(50.8)	
Others	27	15(55.6)	12(44.4)	
Concurrent CRT				
Yes	253	146(57.7)	107(42.3)	0.281
No	75	38(50.7)	37(49.3)	
T stage				
T_1–2_	187	107(57.2)	80(42.8)	0.637
T_3–4_	141	77(54.6)	64(45.4)	
N stage				
N_0–1_	64	42(65.6)	22(34.4)	0.087
N_2–3_	264	142(53.8)	122(46.2)	
PTV volume				
≤ 330 cc	164	95(57.9)	69(42.1)	0.504
>330 cc	164	89(54.3)	75(45.7)	
Lung volume				
≤ 3248 cc	177	101(57.1)	76(42.9)	0.703
>3248 cc	151	83(55.0)	68(45.0)	
V_5_				
≤ 44	160	95 (59.4)	65 (40.6)	0.243
>44	168	89 (53.0)	79 (47.0)	
V_20_				
≤ 24	187	114 (61.0)	73 (39.0)	0.041
>24	141	70 (49.6)	71 (50.4)	
V_30_				
≤ 14.2	122	71 (57.4)	52 (42.6)	0.691
>14.2	205	113 (55.1)	92 (44.9)	
MLD				
≤ 1226 cGy	164	106 (64.6)	58 (35.4)	0.002
>1266 cGy	164	78 (47.6)	86 (52.4)	
RP				
1	262	137(52.3)	125(47.7)	0.006
2	49	32(65.3)	17(34.7)	
3–4	17	15(88.2)	2(11.8)	

*CRT* Chemoradiotherapy, *MLD* Meal lung dose, *RP* Radiation Pneumonitis

### RP

Of all patients included in this study, 66 patients (20.1%) developed grade ≥ 2 RP, 49 (14.9%) grade 2 and 17 (5.2%) grade 3 or 4. In Pearson’s χ^2^ test, the gemcitabine group experienced a higher incidence of grade ≥ 2 RP, compared with docetaxel group (25.5% Vs. 13.2%, *P* = 0.005), shown in Table 1.

### Determination of the optimal cut-off value of DVH variables

**Fig. 1:**
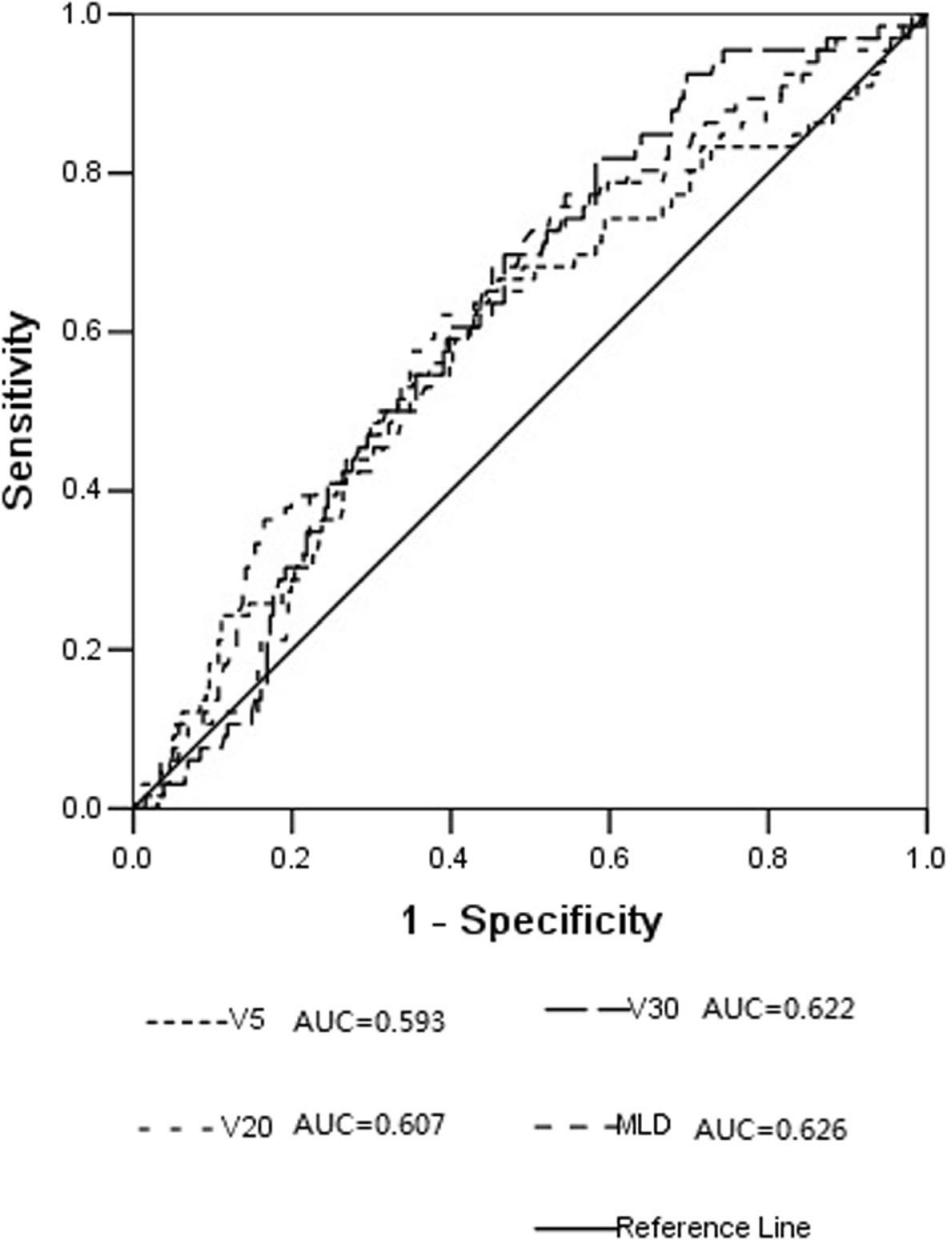
Receiving operator characteristic curve based on the sensitivity and specificity of lung V_5_, V_20_, V_30,_ and MLD

ROC analysis was performed, and the curves with AUCs were shown in Fig. 1. The AUCs for lung V_5_, V_20_, V_30,_ and MLD are all statistically significant (*P*<0.05). After calculation the highest Youden index, the optimal cutoff values of lung V_5_, V_20_, V_30_ and MLD were set at 44% (AUC = 0.593), 24% (AUC = 0.607), 14.2% (AUC = 0.622) and 1226 cGy (AUC = 0.626). Enrolled patients were stratified into high or low level by various DVH variables based on ROC analysis.

### Risk factors for grade ≥ 2 RP

On univariate analysis, gemcitabine chemotherapy was associated with 2.26-fold elevated risk for grade ≥ 2 RP (95% CI: 1.26–4.05, *P* = 0.006), compared with docetaxel chemotherapy. Then we investigated the risk factors for RP in the gemcitabine group. Lung V_5_, V_20_, V_30,_ and MLD were all associated with increased risk of grade ≥ 2 RP in univariate analysis (Table 2). To find the independent risk factors, a multivariate logistic regression model was built. On multivariate analysis, only lung V_30_ was identified as a predictor for grade ≥ 2 RP (*P* = 0.03, shown in Table 3). Compared with low lung V_30_ (≤14.2%), high V_30_ (>14.2%) was associated with 2.92-fold increased risk of RP.

**Table 2:** Association of RP with dose-volume variables

	Gemcitabine				Docetaxel			
Variable	n	Grade ≥ 2 RP, n(%)	Hazard Ratio	*P*	n	Grade ≥ 2 RP, n(%)	Hazard Ratio	*P*
Lung V_5_								
≤ 44	95	14	1	0.001	65	8	1	0.781
>44	89	33	3.41 (1.67–6.95)		79	11	1.15 (0.43–3.06)	
Lung V_20_								
≤ 24	114	18	1	<0.001	73	7	1	0.200
>24	70	29	3.77 (1.89–7.54)		71	12	1.92 (0.71–5.19)	
Lung V_30_								
≤ 14.2	71	9	1	0.002	52	4	1	0.151
>14.2	113	38	3.49 (1.57–7.77)		92	15	2.34 (0.73–7.46)	
MLD								
≤ 1226 cGy	106	17	1	0.001	58	4	1	0.076
>1226 cGy	78	30	3.27 (1.64–6.53)		86	15	2.85 (0.90–9.08)

**Table 3:** Multivariable analysis for RP ≥ 2 in patients treated with gemcitabine

Factors	Multivariable analysis		
	HR	95% CI	*P*
Lung V_5_ >44 Vs ≤44	1.87	0.82–4.27	0.14
Lung V_20_ >24 Vs ≤24	1.68	0.59–4.80	0.33
Lung V_30_ >14.2 Vs ≤14.2	2.92	1.10–7.74	0.03
MLD >1226 cGy Vs ≤1226 cGy	0.49	0.15–1.56	0.23

### RP-free survival

**Fig. 2:**
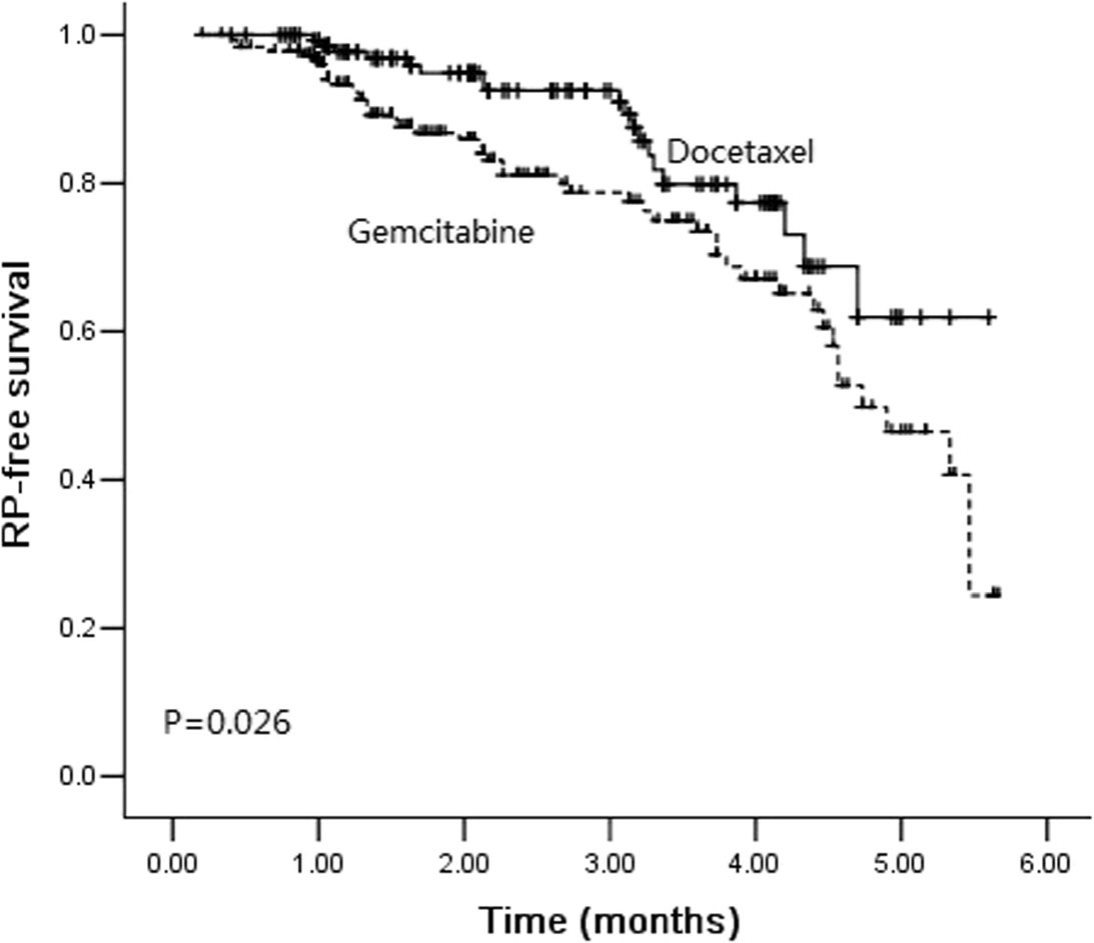
Kaplan-Meier estimates of radiation pneumonitis-free survival between gemcitabine group and docetaxel group

In the whole cohort, patients treated with gemcitabine was associated with a shorter interval of RP-free survival when compared with those treated with docetaxel (Fig. 2, *P* = 0.026). In the group of gemcitabine, RP-free survival did not differ by patients gender (*P* = 0.612). Neither total lung volume, nor PTV volume was significantly differenced for RP-free survival (*P*>0.05). Moreover, patients’ age, T stage, and N stage did not seem to have an impact on RP-free survival (*P*>0.05). All DVH parameters, such as lung V_5_, V_20_, V_30,_ and MLD, were relevant to RP-free survival (*P*<0.05).

**Fig. 3:**
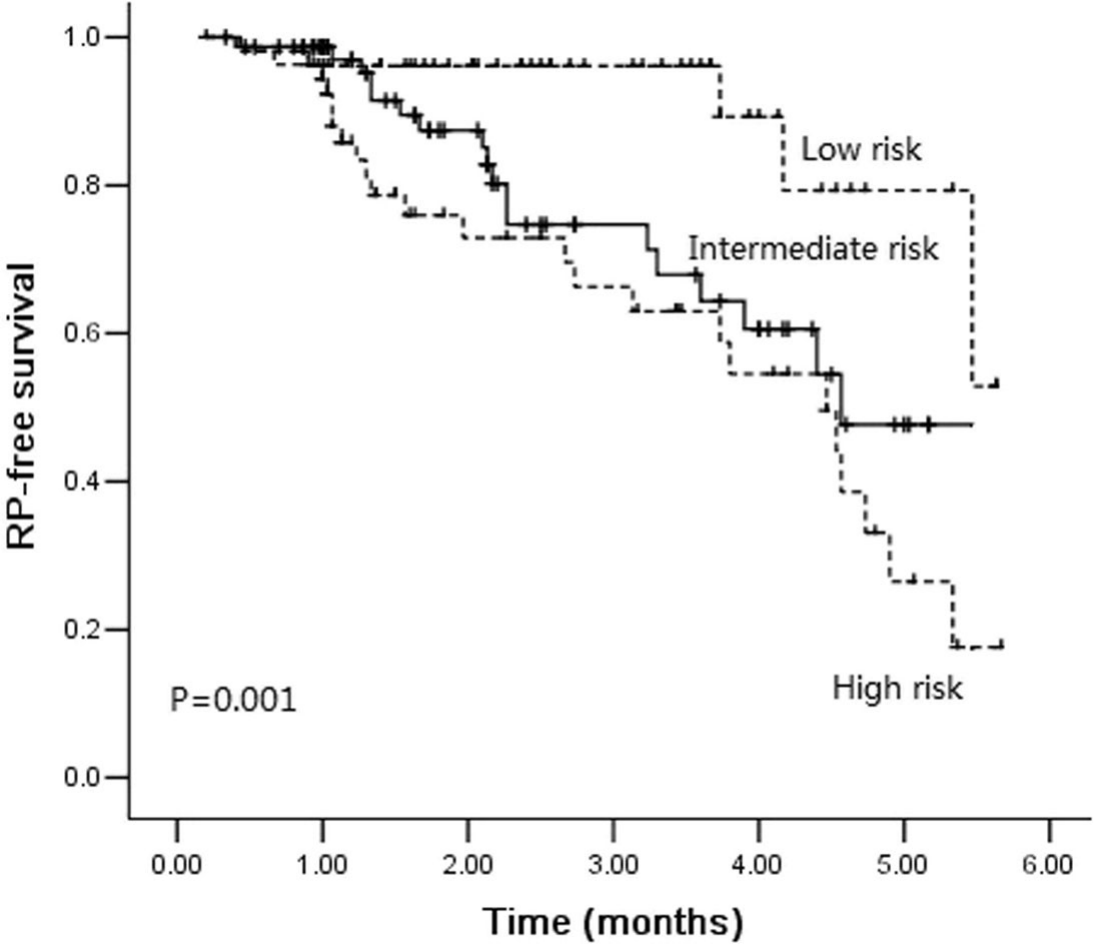
Kaplan-Meier estimates of radiation pneumonitis-free survival among different risk groups

Furthermore, we created a risk model that comprised the following factors: lung V_5_ (score 0 when ≤44%, 1 when >44%), lung V_20_ (score 0 when ≤24%, 1 when >24%), lung V_30_ (score 0 when ≤14.2%, 1 when >14.2%) and MLD (score 0 when ≤1226 cGy, 1 when >1226 cGy). Then we grouped patients according to the sum of above scores: the high-risk group, patients with a score of 4; the intermediate-risk group, patients with a score of 1–3; the low-risk group, patients with a score of 0. Remarkably, the low-risk group was associated with a longer interval of RP-free survival (Fig. 3, *P* = 0.001). The grade ≥ 2 RP rate was 9.4% for the low-risk group (5 of 53 patients), 24.7% for the intermediate group (19 of 77 patients) and 42.6% for the high-risk group (23 of 54 patients).

## Discussion

In this study, gemcitabine chemotherapy before thoracic radiotherapy in NSCLC patients was related to a higher incidence of grade ≥ 2 RP, compared with docetaxel chemotherapy. The frequency of RP varies with the use of different chemotherapeutic drugs during thoracic radiotherapy [16]. The risk of RP following early chemotherapy drugs combined with radiotherapy such as bleomycin, procarbazine, and ifosfamide was 38, 26, and 20%, respectively. Because of the high toxicities of treatment, these drugs were gradually replaced by a new generation of drugs with low toxicities. Subsequent clinical observations also confirmed the relatively low pulmonary toxicities of cisplatin [17], carboplatin, and paclitaxel [1]. The incidence of RP after pemetrexed based concurrent chemoradiotherapy was only 1.8%, according to PROCLAIM study [18]. Gemcitabine has been shown to have efficacy as a single agent or in combination with other chemotherapeutic agents for lung cancer, especially lung squamous cell carcinoma [19]. However, its pulmonary toxicity limits widespread use, especially in combination with thoracic irradiation [20]. In the present study, 25.5% of patients who received gemcitabine chemotherapy before radiotherapy developed grade ≥ 2 RP. This result was comparable to the 22% which was found by Kosmidis P et al. [21].

Many studies have demonstrated that gemcitabine-based chemoradiotherapy was active, with a satisfactory prognosis. However, its pulmonary toxicity was intolerable. The RP occurrence in NSCLC patients who received gemcitabine-based induction chemotherapy has been studied in several publications (Shown in Table 4) [10, 21–28]. The rate of grade ≥ 3 RP in patients who received sequential radiotherapy after gemcitabine-based induction chemotherapy was about 15% [22, 24]. When patients with locally advanced NSCLC treated with concurrent radiotherapy and low dose gemcitabine after induction chemotherapy with gemcitabine, the frequency of grade ≥ 3 RP was 35% [23, 25, 26]. Arrieta et al. [26] treated patients with stage IIIA or IIIB NSCLC using gemcitabine (800 mg/m^2^) and carboplatin (area under the curve [AUC] = 2.5) every 3 weeks for two cycles, followed by conventional fraction radiotherapy to 60 Gy combined with weekly gemcitabine (200 mg/m^2^). That study was closed due to excessive pulmonary toxicity. 6 of 19 patients developed grade ≥ 3 RP, and one patient died of uncontrollable RP. Furthermore, when the total dose of radiotherapy increased to 74 Gy, the incidence of fatal RP was as high as 37% [25]. These results suggested that induction chemotherapy using gemcitabine was needed careful consideration in term of predictable pulmonary toxicity. In our present study, the rate of grade ≥ 3 RP in patients received gemcitabine induction chemotherapy was 5.3%. This number was lower than that in the studies listed above. In our study, most patients received concurrent chemoradiotherapy with paclitaxel and carboplatin. Furthermore, the dose of thoracic radiotherapy was 60 Gy (Standard dose) instead of 74 Gy (High dose), according to the result of RTOG 0617 [29]. Also, the use of IMRT technique for locally advanced NSCLC in our study was associated with lower rates of severe RP [30].

**Table 4:** The RP occurrence in NSCLC patients treated with gemcitabine-based induction chemotherapy

Authors	Year	N	IC	Cycles of IC	RT dose (Gy)	CCRT	RP (Grade, %)	Ref
Guilbault C	2017	142	Gemcitabine+ Cisplatin	2	60	Gemcitabine+ Cisplatin	≥3, 10%	10
Kosmidis P	2007	43	Gemcitabine+Paclitaxel	2	63	Paclitaxel	≥2, 22%	21
Schallier D	2009	64	Gemcitabine+Carboplatin+ Paclitaxel	3	66	–	≥3, 12.5%	22
Blanco R	2008	56	Gemcitabine+Cisplatin	3	68.4	Gemcitabine	≥3, 34%	23
Belderbos J	2007	78	Gemcitabine+Cisplatin	2	66	–	≥3, 14%	24
Socinski MA	2008	26	Gemcitabine+Carboplatin	2	74	Gemcitabine	≥3, 37%	25
Arrieta O	2009	19	Gemcitabine+Carboplatin	2	60	Gemcitabine	≥3, 31.6%	26
Kocak M	2009	39	Gemcitabine+Cisplatin	3	66	Docetaxel+cisplatin	NR. 31%	27
Driesen P	2013	49	Gemcitabine+ Cisplatin	3	63	Gemcitabine+ Cisplatin	≥3, 6.5%	28

*IC* Induction chemotheraoy, *RT* Radiotherapy, *CCRT* Concurrent chemoratiotherapy, *RP* Radiation pneumonitis, *NR* Not reffered, *Ref* Reference

Taxanes, such as paclitaxel and docetaxel, were common chemotherapeutic regimens with high efficacy in NSCLC. Comparison of taxanes based and gemcitabine bases chemotherapy has been performed in several studies. In the clinical trial CALGB 30105, patients with locally advanced NSCLC were randomly assigned to induction chemotherapy with either paclitaxel with carboplatin or gemcitabine with carboplatin followed by thoracic radiotherapy (74 Gy). The rate of grade ≥ 3 RP were 16.2 and 39.1% in the paclitaxel group and gemcitabine group, respectively. The rate of RP was significantly higher in patients who received gemcitabine induction chemotherapy than that in patients received paclitaxel (*P* = 0.046). In a retrospective study [27], the rate of RP for gemcitabine induction group was higher than that for docetaxel induction group (31.0% Vs. 20.8%) but did not reach the significant difference (*P* = 0.314). In our study, compared with docetaxel group, we found a higher incidence of grade ≥ 3 RP in the gemcitabine group (25.5% Vs. 13.2%, *P* = 0.005). Furthermore, RP occurred earlier in the gemcitabine group than that in the docetaxel group (*P* = 0.026). The results of this study suggested that gemcitabine-based induction chemotherapy, followed by radiotherapy, was associated with a high incidence of RP. Modification of gemcitabine dosage or cycles before radiotherapy or limitation in radiotherapy planning might reduce pulmonary toxicity.

The lung is composed of massive parallel functional subunits. Even if some subunits were damaged by X-rays, the function of others could still operate normally. Therefore, the incidence of RP is related to the volume of the lung exceeding the lung radio-tolerant dosage. Numerous studies demonstrated that dose-volume parameters, such as lung V_5_, V_10_, V_20_, V_30_, V_50,_ and MLD, could efficiently predict the occurrence of RP. In our study, all dosimetric parameters considered in the study (lung V_5_, V_20_, V_30_, and MLD) correlated significantly with RP in all patients and in the subgroup of patients who received gemcitabine induction chemotherapy. On multivariate analysis, only lung V_30_ was identified as an independent predictor for grade ≥ 2 RP, findings that correspond with those from several publications [31–33]. The results of Hernando ML et al.’ study [34] showed that V_30_ ≥ 18% was one of the best predictors of severe acute RP among patients treated with definitive external beam radiotherapy. The observed incidence of RP was 24% for patients with V_30_ ≥ 18 and 6% for those with V_30_<18%. The cut-off value of lung V_30_ in our study was set as 14.2% according to the calculation the highest Youden index, findings that were slightly different from other studies. The incidence of RP was as high as 33.6% for patients with V_30_ ≥ 14.2 and 12.9% for patients with V_30_<14.2%. The cut-off values of lung V_30_ in patients treated with concurrent chemoradiotherapy [31] or adjuvant radiotherapy after lobectomy [35] were 22 and 13%, respectively. Furthermore, we created a risk model based on all dosimetric parameters considered in the study. The RP rate was less than 10% when all dosimetric parameter’s constraints were satisfied.

However, there were some limitations in this study. Selective bias still exists because it was a retrospective study. Secondly, radiation-induced pulmonary toxicities included radiation pneumonitis and pulmonary fibrosis. In the present study, only the occurrence and risk factors of RP were analyzed. Furthermore, because most of the patients included in this study were with lung squamous cell carcinoma, there were more male patients and fewer female patients. This limitation made the conclusions less definitive.

## Conclusion

In conclusion, this study found that gemcitabine chemotherapy before thoracic radiotherapy in NSCLC patients was related to a higher incidence of grade ≥ 2 RP, compared with docetaxel chemotherapy. Lung V_5_, V_20_, V_30,_ and MLD were all associated with increased risk of grade ≥ 2 RP in patients received gemcitabine induction chemotherapy followed by radiotherapy. We also created a risk model that could be used in clinical practice. The grade ≥ 2 RP rate was only 9.4% for the low-risk group (Lung V_5_ ≤ 44%, V_20_ ≤ 24%, V_30_ ≤ 14.2%, and MLD ≤ 1226 cGy). These dosimetric parameters are recommended for NSCLC patients who received gemcitabine chemotherapy before thoracic radiotherapy.

## Data Availability

All data generated or analysed during this study are included in this published article.

## Abbreviations

ADL: Activities of daily living
AUC: Area under curves
CTCAE: Common Terminology Criteria for Adverse Events
CTV: Clinical tumor volume
DVH: Dose-volume histogram
GTV: Gross tumor volume
IASLC: International Association for the Study of Lung Cancer
MLD: Mean lung dose
NCCN: National Comprehensive Cancer Network
NSCLC: Non-small cell carcinoma
OAR: Organs at risk
PTV: Planning target volume
ROC: Receiver operating characteristic
RP: Radiation pneumonitis
